# Relationship Between Physical Activity and Visual Acuity in Japanese Students: A Cross‐Sectional Study

**DOI:** 10.1002/hsr2.70632

**Published:** 2025-04-07

**Authors:** Kensaku Sasayama

**Affiliations:** ^1^ Faculty of Education Mie University Mie Japan

**Keywords:** accelerometry, exercise, myopia, visual acuity

## Abstract

**Background and Aims:**

Physical activity, including outdoor activities, has been assessed using questionnaires or accelerometers. Examining the relationship between physical activity and myopia is important for future intervention studies. This study aimed to evaluate physical activity using a widely used questionnaire and an accelerometer and to examine their relationship with myopia.

**Methods:**

In Study 1, physical activity was assessed using a questionnaire in 613 primary school students (Grades 3–6, aged 8–12) and 438 secondary school students (Grades 1–3, aged 12–15). In Study 2, physical activity in 55 primary school students (Grades 3–6, aged 8–12) was measured using an accelerometer. Visual acuity was determined using Landolt ring tests. Screen time was also assessed in both studies. The relationship between physical activity (independent variable) and visual acuity (dependent variable) was analyzed using binomial logistic regression, adjusting for grade, gender, and screen time.

**Results:**

No significant association was found between visual acuity and physical activity as measured by either questionnaire or accelerometer in both primary and secondary students, even after adjustments for grade, gender, and screen time.

**Conclusion:**

Physical activity measured by questionnaires and accelerometers did not show an association with myopia. However, this does not exclude a potential association between outdoor activity and myopia. Our findings suggested that these methods of assessing physical activity may not be suitable proxies for outdoor activity in myopia research.

## 1 Introduction

The global population affected by myopia is estimated to reach between 1.4 and 4.8 billion by 2050, with its prevalence expected to rise significantly [1]. Increasing rates of myopia in children have been reported across Asia, Europe, and North America [2]. In East Asia, particularly, the prevalence of myopia has surged over the past 60 years. For example, the estimated prevalence of myopia among 20‐year‐olds in Hong Kong, Taiwan, Singapore, and South Korea was around 20% in 1950, but this figure rose to approximately 80% by 2010 [3]. A similar trend is observed in Japan, where national surveys show that the proportion of high school students with “naked eye vision less than 1.0” increased from 51.6% in 1985 to 71.6% in 2022 [4].

Genetic and environmental factors, including parental myopia, near work (including screen time), lifestyle, light exposure, and population density, are complexly related to myopia development. Notably, outdoor activities have been suggested as a potential preventive factor against myopia [2, 5]. Physical activity, including outdoor activity, is commonly assessed using questionnaires. Among these, the World Health Organization's Health Behavior in School‐aged Children (WHO HBSC) questionnaire is widely used internationally as a subjective assessment tool for physical activity [6].

Accelerometers, such as the ActiGraph, offer a more reliable, valid, and objective measure of physical activity levels. ActiGraph is frequently used in international studies, with over half (51%) of studies assessing physical activity in adults using this device across 76 research projects from 36 countries [7]. Although accelerometers have limitations in that they cannot accurately assess activities carried out underwater and while cycling, they have been validated for assessing physical activity in daily life [8]. In pediatric populations, ActiGraph is also widely adopted, as seen in studies from nine countries [9] and 15 countries [10].

Although the WHO HBSC questionnaire and the ActiGraph accelerometer are widely used and validated for assessing physical activity, they may not be suitable for assessing physical activity specifically related to outdoor activity, which is considered to reduce myopia. Suhr Thykjær et al. [11] highlighted the challenges of not distinguishing between general physical activity and outdoor activity. In their review [11] of the relationship between myopia and physical activity, they found that six out of nine studies reported a negative correlation between physical activity and myopia, with three additional studies supporting this association. However, their findings suggested that outdoor activity, rather than physical activity itself, was associated with myopia. One study even found no association between physical activity and myopia.

Furthermore, Suhr Thykjær et al. [11] emphasized the need for objective methods such as accelerometers to measure physical activity accurately. However, previous studies that used accelerometers to assess physical activity in relation to myopia have produced mixed results. While Guggenheim et al. [12] found a negative association between physical activity and myopia, two other studies observed no such association [13, 14]. Examining the relationship between physical activity and myopia is important for future intervention studies. Therefore, this study aimed to assess physical activity using both the WHO HBSC questionnaire and the ActiGraph accelerometer and to examine their relationship with myopia. The hypothesis was that physical activity, as measured by these tools, would not be associated with myopia.

## 2 Materials and Methods

### 2.1 Ethics Statement

This study was approved by the Institutional Review Board of Mie University (Approval Nos. 2021‐03 and 2022‐01). Written informed consent was obtained from all participating parents before the study. The study procedure was explained to all participants and their parents, who were given the opportunity to opt‐out at any time.

### 2.2 Study Design and Participants

#### 2.2.1 Study 1

Study 1, conducted in 2022, was a cross‐sectional survey assessing visual acuity, physical activity, and screen time among primary and secondary school students in the Isshinden area of Mie Prefecture, Japan. This study included all three primary schools (Grades 3–6, 640 students, aged 8–12) and 1 secondary school (Grades 1–3, 469 students, aged 12–15). This study covered all children enrolled in school, as the study was integrated into the school curriculum, it achieved a 100% participation rate.

#### 2.2.2 Study 2

Study 2, also conducted in 2022, was a cross‐sectional survey of visual acuity, physical activity, and screen time among primary school students in Okayama Prefecture, Japan. This study covered all children enrolled in school. This study involved 61 students in Grades 3–6 from one primary school in Okayama City, with a participation rate of 33.9% (61 of 180 students).

### 2.3 Visual Acuity Assessment

#### 2.3.1 Study 1 and Study 2

Visual acuity was assessed using standardized tests conducted at each school, as described in a previous nationwide study in primary and secondary schools [15]. The tests used a Landolt ring at a distance of 5 m from the optotype to the eye, with students either standing or sitting. The height of the eye and the optotype was matched, and the optotype was presented perpendicular to the gaze. For the test, the left eye was occluded with an eye shield to prevent pressure, and students were asked to identify the direction of the slit in the Landolt ring with their right eye. Subsequently, the same procedure was repeated for the left eye. Testing began with the 0.3 optotype, presented in any of four directions (up, down, left, right) for 3–5 s. If the participants correctly identified the direction in two or fewer trials, they were classified as “not discriminative” and assigned a “D.” If they correctly identified three directions, they were classified as “correctly discriminative,” and the left eye was tested. If they could not correctly identify the directions in the 0.7 optotype, the students were assessed as having a “C.” If correct discrimination was made, they moved on to the 1.0 optotype. If they could not correctly identify the directions in the 1.0 optotype, they were assessed as having made a “B.” If correct discrimination was made, they were assessed as having an “A.” Visual acuity categories were defined as follows: A (≥ 1.0), B (0.9–0.7), C (0.6–0.3), and D (< 0.3). Students who used glasses, contact lenses, or orthokeratology were categorized as “E.” If visual acuity differed between the eyes, the lower value was recorded. For this study, participants were divided into two categories: A and the others (B, C, D, E).

### 2.4 Physical Activity Assessment

#### 2.4.1 Study 1

Physical activity was assessed through moderate‐to‐vigorous physical activity (MVPA) using the Japanese version of the physical activity questions in the WHO HBSC (WHO HBSC‐J) questionnaire, as reported by Tanaka et al. [16, 17]. The key question was “Over the past 7 days, on how many days were you physically active for a total of at least 60 min per day?” Participants responded with a number ranging from 0 to 7 days. Guidelines for children and adolescents aged 5–17 years emphasize the importance of an average of 60 min of MVPA daily [18]. Given that previous studies [16, 17] reported that few children achieve 60 min of MVPA every day for 7 days, participants in Study 1 were divided into a high group and a low group based on whether they engaged in 60 min of MVPA on five or more days.

#### 2.4.2 Study 2

In Study 2, physical activity was assessed using MVPA measured by an ActiGraph GT9X Link accelerometer (ActiGraph LLC, Pensacola, FL, USA). Participants wore the accelerometer on their waist continuously for seven consecutive days, including both weekdays and weekends, except during sleep, swimming, bathing, or contact sports. Data were recorded in 15‐second epochs, with valid data defined as a minimum of 4 days of wear time, each with at least 480 min of valid data (excluding intervals of 30 min with zero counts). Sedentary time, LPA, and MVPA were calculated using cutoffs established by Evenson et al. [8]. In Study 2, participants were categorized into high and low groups based on whether they averaged 60 min of MVPA per day, in accordance with physical activity guidelines for children [18].

### 2.5 Screen Time Assessment

#### 2.5.1 Study 1

Screen time was assessed using a questionnaire that asked participants about their daily time spent on various activities. The questions included “How much time do you spend reading books, newspapers, magazines, or comics outside of school on a daily basis?”, “How many hours do you play nononline games daily?”, “Outside of school, how much time do you spend watching TV, videos, or DVDs (excluding online videos) daily?”, “How much do you use your mobile phone/smartphone or tablet/computer outside of school daily?”, and “On average, how much do you study outside of school daily?” In Study 1, screen time was calculated by summing the response to these questions.

#### 2.5.2 Study 2

In Study 2, screen time was assessed using a questionnaire that included the following items: “How much time per day do you usually spend watching TV, videos, and DVDs (including YouTube)?”, “How many hours per day do you usually play games (excluding physical games)?”, and “How much time per day do you usually spend on the internet using a computer, tablet, or smartphone?”

Participants selected from the following options: “not at all,” “about 30 min,” “about 1 h,” “about 2 h,” “about 3 h,” “about 4 h,” “about 5 h,” “about 6 h,” and “about 7 h or more,” for both weekdays and weekends. Weekly screen time was calculated by averaging the total weekday hours (multiplied by 5) and the total weekend hours (multiplied by 2), then dividing by 7. In Study 2, screen time was defined as the total time spent per day on each question.

### 2.6 Statistical Analyses

Participant characteristics, visual acuity, physical activity, and screen time variables are presented as mean ± standard deviation or *n* (%). The relationship between physical activity (independent variable) and visual acuity (dependent variable) was analyzed using binomial logistic regression, adjusted for grade, gender, and screen time. The odds ratio represents the likelihood of having visual acuity less than 1.0. All analyses were conducted using the IBM SPSS Statistics software package version 29.0 (IBM Japan, Tokyo, Japan), with statistical significance set at *p* < 0.05.

## 3 Results

### 3.1 Participant Characteristics

Table 1 presents the characteristics of participants in Study 1 and Study 2. After excluding those with missing data, 613 primary school students and 438 secondary school students were analyzed in Study 1 and 55 primary school students were analyzed in Study 2.

**Table 1:** Characteristics of the participants.

	Study 1		Study 2
	Primary school students	Secondary school students	Primary school students
	(*n* = 613)	(*n* = 438)	(*n* = 55)
	Mean ± SD or *n* (%)	Mean ± SD or *n* (%)	Mean ± SD or *n* (%)
Age (years)	9.6 ± 1.1	13.0 ± 0.8	10.0 ± 1.1
Gender (boys)	305 (49.8)	231 (52.7)	31 (56.4)
Visual acuity			
A (> 1.0)	280 (45.7)	177 (40.4)	39 (70.9)
B (0.7–0.9)	77 (12.6)	28 (6.4)	4 (7.3)
C (0.3–0.6)	64 (10.4)	58 (13.2)	5 (9.1)
D (< 0.2)	56 (9.1)	12 (2.7)	5 (9.1)
E (vision correction)	136 (22.2)	163 (37.2)	2 (3.6)
Physical activity			
Questionnaire			
Day of MVPA at least 60 min/day			
0 (day/week)	61 (10.0)	70 (16.0)	—
1 (day/week)	42 (6.9)	29 (6.6)	—
2 (day/week)	71 (11.6)	33 (7.5)	—
3 (day/week)	64 (10.4)	52 (11.9)	—
4 (day/week)	64 (10.4)	50 (11.4)	—
5 (day/week)	116 (18.9)	90 (20.5)	—
6 (day/week)	57 (9.3)	38 (8.7)	—
7 (day/week)	138 (22.5)	76 (17.4)	—
Accelerometer			
MVPA (min/day)	—	—	58.9 ± 27.9
Number of students meeting 60 min MVPA	—	—	24 (43.6)
Screen time^a^ (min/day)	478.3 ± 362.0	556.0 ± 339.9	323.5 ± 226.7

Abbreviations: SD, standard deviation; VPA, moderate‐to‐vigorous physical activity.

^a^ Study 1 and Study 2 have different questions.

### 3.2 Relationship Between Visual Acuity and Physical Activity Assessed by Questionnaire

Table 2 shows the relationship between visual acuity and physical activity assessed by questionnaire in Study 1. No association was observed between visual acuity and physical activity in either primary or secondary schools. This result remained consistent after adjusting for grade, gender, and screen time.

**Table 2:** Relationship between visual acuity and physical activity assessed by questionnaire.

		Visual acuity	
		Model 1 (unadjusted)	Model 2 (adjusted)
		OR (95% CI)	OR (95% CI)
Primary school students			
Physical activity	High (*n* = 311)	1.00 (reference)	1.00 (reference)
	Low (*n* = 302)	0.92 (0.67–1.27)	0.90 (0.65–1.24)
Secondary school students			
Physical activity	High (*n* = 204)	1.00 (reference)	1.00 (reference)
	Low (*n* = 234)	1.39 (0.95–2.03)	1.26 (0.85–1.87)

*Note:* The high group includes those who engage in 60 min of moderate‐to‐vigorous physical activity on at least five days a week, while the low group includes those who do less than five days a week. Analyses were adjusted for grade, gender, and screen time. The table shows the OR representing the relationship between physical activity and visual acuity of less than 1.0.

Abbreviations: CI, confidence interval; OR, odds ratio.

### 3.3 Relationship Between Visual Acuity and Physical Activity Assessed by Accelerometer

Table 3 presents the relationship between visual acuity and physical activity assessed by accelerometer in Study 2. No association was observed between visual acuity and physical activity assessed by accelerometer in primary schools. This result remained consistent after adjusting for grade, gender, and screen time.

**Table 3:** Relationship between visual acuity and physical activity assessed by accelerometer.

		Visual acuity	
		Model 1 (unadjusted)	Model 2 (adjusted)
		OR (95% CI)	OR (95% CI)
Primary school students			
Physical activity	High (*n* = 24)	1.00 (reference)	1.00 (reference)
	Low (*n* = 31)	1.43 (0.43–4.71)	1.51 (0.42–5.37)

*Note:* The high group includes those students who met the 60 min of moderate‐to‐vigorous physical activity per day, while the low group includes those who did not. Analyses were adjusted for grade, gender and screen time. The table shows the OR representing the relationship between physical activity and visual acuity of less than 1.0.

Abbreviations: CI, confidence interval; OR, odds ratio.

## 4 Discussion

This study examined the association between physical activity, assessed by both questionnaire and accelerometer, and myopia in primary and secondary school children. The findings revealed no association between physical activity and myopia, regardless of the assessment methods. These results indicate that physical activity, as assessed by questionnaires and accelerometers—commonly used internationally for assessing outdoor activity—may not be a suitable indicator for evaluating the risk of myopia.

In this study, no association was observed between physical activity assessed by the questionnaire and visual acuity (Table 2). Previous studies that reported an association between physical activity and myopia using questionnaires found negative correlations [19, 20]. Physical activity, defined as “any bodily movement produced by skeletal muscles that results in energy expenditure” [21] includes all daily activities, both indoors and outdoors. However, it has been noted that exposure to daylight through outdoor activities is necessary to effectively reduce the risk of myopia [22]. Therefore, the differences between the results of this study and previous studies may be partly due to the inadequate assessment of outdoor activities.

Similar to the questionnaire findings in this study, no association was observed between physical activity assessed by accelerometer and visual acuity (Table 3). Three studies have examined the relationship between physical activity measured by accelerometers and visual acuity, with two of these studies [13, 14] finding no association. Our findings align with these two studies, suggesting no association between physical activity assessed by accelerometer and visual acuity. Guggenheim et al. [12] investigated the relationship between incident myopia and both time spent outdoors and physical activity, as measured separately by accelerometer. Their results indicated that while both time spent outdoors and physical activity were associated with incident myopia, time spent outdoors had a more significant impact. They also found that time spent outdoors was a predictor of incident myopia, independent of physical activity levels. These findings suggested that assessing physical activity using questionnaires and accelerometers, as commonly used internationally, may not be an appropriate substitute for measuring outdoor activity. Future research examining the association between myopia and outdoor activity should assess outdoor activity separately from physical activity or use an illuminance meter. Additionally, the effects of physical activity or outdoor activity on myopia should be further investigated through randomized controlled trials.

### 4.1 Limitations

This study has several limitations. First, it has been reported that the score for the Landolt ring test is lower than that for other tests (e.g., Early Treatment of Diabetic Retinopathy Study chart [23], E tumbling test [24]). In addition, in this study, the Landolt ring was used to assess uncorrected visual acuity, so there is a limit to the extent to which myopia can be assessed accurately. Furthermore, this study classified the results into A (≥ 1.0) and the others (B, C, D, E), but the groups other than A may include hyperopia and astigmatism. Therefore, the fact that refractive errors were not assessed correctly is a major limitation of this study. Second, the WHO HBSC‐J was validated for grades 5 and 6 of primary school and Grades 1–3 of secondary school, whereas this study included students in Grades 3 and 4 of primary school for whom the validity of the questionnaire has not been confirmed. This may have introduced some bias into the results. Third, only a small number of primary school children were included in the accelerometer survey (Study 2), and no secondary school students were not assessed by accelerometers. Fourth, while this study adjusted for screen time, an associated factor for myopia, we did not consider other genetic factors, such as parental myopia, which is a limitation of this study. Finally, as this is a cross‐sectional study, it is not possible to make any statements about cause and effect. Therefore, it will be necessary to conduct longitudinal studies of physical activity and visual acuity in the future.

## 5 Conclusion

In this study, physical activity assessed by both questionnaire and accelerometer did not show any association with myopia. However, these results do not rule out a potential association between outdoor activity and myopia. The findings suggest that it is crucial to accurately assess outdoor activity when examining its association with myopia in future studies or when conducting intervention studies aimed at promoting outdoor activity to prevent myopia.

## Author Contribution

**Kensaku Sasayama:** conceptualization, methodology, investigation, formal analysis, funding acquisition, writing – original draft, writing – review and editing.

## Conflicts of Interest

The author declares no conflicts of interest.

## Transparency Statement

The lead author Kensaku Sasayama affirms that this manuscript is an honest, accurate, and transparent account of the study being reported; that no important aspects of the study have been omitted; and that any discrepancies from the study as planned (and, if relevant, registered) have been explained.

## Data Availability

The data that support the findings of this study are available from the corresponding author upon reasonable request.
